# A new species of *Otacilia* Thorell, 1897 (Araneae, Phrurolithidae) from Tongboshan National Nature Reserve, Jiangxi Province, China

**DOI:** 10.3897/BDJ.13.e144804

**Published:** 2025-01-28

**Authors:** Zimin Jiang, Changyong Lin, Zhongjing Wang, Yanbin Yao, Keke Liu

**Affiliations:** 1 College of Life Science, Jinggangshan University, Ji'an, China College of Life Science, Jinggangshan University Ji'an China; 2 Tongboshan National Natrue Reserve, Shangrao, China Tongboshan National Natrue Reserve Shangrao China; 3 Jinshan College of Fujian Agriculture And Forestry University, Fuzhou, China Jinshan College of Fujian Agriculture And Forestry University Fuzhou China

**Keywords:** Sac spiders, taxonomy, phrurolithid species

## Abstract

**Background:**

Sixty-four phrurolithid species were found from Jiangxi Province in the past five years. However, there are still many unknown phrurolithid species from this Province with unusual morphological characteristics.

**New information:**

A new species, *Otaciliatongboshan* Liu, **sp. nov.** is described from Tongboshan National Nature Reserve, Jiangxi Province, China. Morphological illustrations, SEMs and living photos and a distribution map are given.

## Introduction

The spider family Phrurolithidae Banks, 1892 is one of the most species-rich families of sac spiders recorded in the world (World Spider Catalog 2024). The knowledge of this richness is increasing greatly year by year (Liu et al. 2020a, Liu et al. 2020b, Liu et al. 2021, Liu et al. 2022, Mu and Zhang 2022, Liu et al. 2023, Mu and Zhang 2023, World Spider Catalog 2024). Currently, there are 406 species from 25 genera worldwide and more than half of them (214 species) belonging to 17 genera have been recorded from China (World Spider Catalog 2024). Most of Chinese phrurolithids are reported from the southern provinces of the country (for references see Liu et al. (2020a), Liu et al. (2020b), Liu et al. (2022), Liu et al. (2023), Mu and Zhang (2023), World Spider Catalog (2024)), such as Anhui (3 species), Guangxi (19 species), Guizhou (8 species), Hainan (11 species), Hubei (9 species), Hunan (30 species), Ningxia (1 species), Shanxi (6 species), Sichuan (21 species), Yunnan (23 species), Zhejiang (7 species), Jiangsu (1 species), Chongqing (17 species) and Jiangxi (64 species). Yet, there still remain many unknown phrurolithid species from southern China with unusual morphological characteristics from our research (Liu et al. 2022).

Although the genus *Otacilia* Thorell, 1897 is the most diverse group amongst these genera, the morphological variation within its supposed members is so broad that the assignment of several species to this genus has been questioned (Zamani and Marusik 2020, Liu et al. 2022, Liu et al. 2023). However, the main difficulty is that *Otacilia* was established by Thorell (1897), based on a single female specimen from Myanmar (Burma). Until now, the male of the type species, *O.armatissima* Thorell, 1897, is still unknown (World Spider Catalog 2024). Therefore, there were many more species wrongly placed in this genus which we have only begun to correct (Liu et al. 2020b, Liu et al. 2022). In the last decade, there have been some significant changes and many species have been newly combined or re-assigned to newly-established genera, such as *Abdosetae* Fu, Zhang & MacDermott, 2010, *Aculithus* Liu & Li, 2022, *Alboculus* Liu, 2020, *Corealithus* Kamura, 2021, *Grandilithus* Liu & Li, 2022, *Lingulatus* Mu & Zhang, 2022, *Lunalithus* Kamura, 2022, *Pennalithus* Kamura, 2021 and *Xilithus* Liu & Li, 2022 (Fu et al. 2010, Liu et al. 2020b, Kamura 2021, Kamura 2022, Liu et al. 2022, Mu and Zhang 2022, Lin et al. 2023, Mu and Zhang 2023). The availability of more taxonomic revisions makes *Otacilia* easier to distinguish from the other genera in the family.

In the past five years, 33 *Otacilia* species have been found from Jiangxi Province (Liu et al. 2020a, Liu et al. 2020b, Liu et al. 2021, Liu et al. 2022, Liu et al. 2023, Mu and Zhang 2023). These results add to a growing body of evidence that the increasing number of *Otacilia* species may predict the high species richness and diversity (Liu et al. 2020a, Liu et al. 2020b, Liu et al. 2021, Liu et al. 2022, Liu et al. 2023). Recently, a major spider investigation was initiated in the Tongboshan National Nature Reserve in Jiangxi Province. When examining the specimens collected from there, one undescribed species was found. The aim of the present paper is to provide a detailed description and figures of this new species.

## Materials and methods

Specimens were examined using a SZ6100 stereomicroscope. Both male and female copulatory organs were dissected and examined in 80% ethanol using an Olympus CX43 compound microscope with a KUY NICE CCD camera. Epigynes were cleared with pancreatin solution (Álvarez-Padilla and Hormiga 2007). For SEM photographs, the specimens were kept under natural dry conditions, coated with gold with a small ion-sputtering apparatus ETD-2000 and photographed with a Zeiss EVO LS15 scanning electron microscope. Types are deposited in the Animal Specimen Museum, College of Life Science, Jinggangshan University (ASM-JGSU).

The measurements were taken using a stereomicroscope (AxioVision SE64 Rel. 4.8.3) and are given in millimetres. The body lengths of all specimens exclude the chelicerae and spinnerets. Terminology of the male and female genitalia follows Liu et al. (2022).

Leg measurements are given as total length (femur, patella, tibia, metatarsus, tarsus). The abbreviations used in the figures and text are as follows: ALE − anterior lateral eye, AME − anterior median eye, Bu – bursa, CD − copulatory duct, CG – cymbial groove, CO − copulatory opening, CT − connecting tube, dTA − distal tegular apophysis, EG – embolic groove, Em – embolus, FA − femoral apophysis, FD − fertilisation duct, GA − glandular appendage, MOA − median ocular area, MS − median septum, PLE − posterior lateral eye, PME − posterior median eye, PP – posterior plate, PTA − prolateral tibial apophysis, rTA − retrolateral tegular apophysis, RTA − retrolateral tibial apophysis, SD − sperm duct, Spe – spermathecae.

## Taxon treatments

### Otacilia tongboshan

Liu sp. nov.

2F41F66C-5D20-53F1-9056-8EB68EB1B707

B145E4B9-E9E5-407F-89D6-C4938C4C881C

#### Materials

- **Type status:** Holotype. **Occurrence:** recordedBy: Liu Ke-Ke; individualCount: 1; sex: male; lifeStage: adult; occurrenceID: 75746E13-942C-5F48-9ED8-101E1D6419D7; **Taxon:** scientificName: *Otaciliatongboshan* sp. nov.; **Location:** country: China; stateProvince: Jiangxi; locality: Shangrao City, Guangfeng District, Tongboshan National Nature Reserve, Xiaocaogou; verbatimElevation: 827 m; verbatimCoordinates: 28°08'52.07"N, 118°13'23.65"E; georeferenceProtocol: GPS; **Event:** samplingProtocol: sieving; eventDate: 30/11/2024
- **Type status:** Paratype. **Occurrence:** recordedBy: Liu Ke-Ke; individualCount: 3; sex: male; lifeStage: adult; occurrenceID: BEF2C66D-FFC8-564C-993C-A052DD996089; **Taxon:** scientificName: *Otaciliatongboshan* sp. nov.; **Location:** country: China; stateProvince: Jiangxi; locality: Shangrao City, Guangfeng District, Tongboshan National Nature Reserve, Xiaocaogou; verbatimElevation: 827 m; verbatimCoordinates: 28°08'52.07"N, 118°13'23.65"E; georeferenceProtocol: GPS; **Event:** samplingProtocol: sieving; eventDate: 30/11/2024
- **Type status:** Paratype. **Occurrence:** recordedBy: Liu Ke-Ke; individualCount: 7; sex: female; lifeStage: adult; occurrenceID: 69592176-A58E-58B3-8DAD-CD1A8E1D7574; **Taxon:** scientificName: *Otaciliatongboshan* sp. nov.; **Location:** country: China; stateProvince: Jiangxi; locality: Shangrao City, Guangfeng District, Tongboshan National Natrue Reserve, Xiaocaogou; verbatimElevation: 827 m; verbatimCoordinates: 28°08'52.07"N, 118°13'23.65"E; georeferenceProtocol: GPS; **Event:** samplingProtocol: sieving; eventDate: 30/11/2024
- **Type status:** Paratype. **Occurrence:** recordedBy: Liu Ke-Ke; individualCount: 5; sex: male; lifeStage: adult; occurrenceID: 19695C27-4DA0-55F7-9FBB-35B3FE48955C; **Taxon:** scientificName: *Otaciliatongboshan* sp. nov.; **Location:** country: China; stateProvince: Jiangxi; locality: Shangrao City, Guangfeng District, Tongboshan National Natrue Reserve, entrance of the Dadongkeng; verbatimElevation: 823 m; verbatimCoordinates: 28°07'40.59"N, 118°12'12.21"E; georeferenceProtocol: GPS; **Event:** samplingProtocol: sieving; eventDate: 30/11/2024
- **Type status:** Paratype. **Occurrence:** recordedBy: Liu Ke-Ke; individualCount: 3; sex: female; lifeStage: adult; occurrenceID: 5910C592-3120-5786-B736-DCA16919A0C2; **Taxon:** scientificName: *Otaciliatongboshan* sp. nov.; **Location:** country: China; stateProvince: Jiangxi; locality: Shangrao City, Guangfeng District, Tongboshan National Natrue Reserve, entrance of the Dadongkeng; verbatimElevation: 823 m; verbatimCoordinates: 28°07'40.59"N, 118°12'12.21"E; georeferenceProtocol: GPS; **Event:** samplingProtocol: sieving; eventDate: 30/11/2024
- **Type status:** Paratype. **Occurrence:** recordedBy: Liu Ke-Ke; individualCount: 2; sex: male; lifeStage: adult; occurrenceID: 4FDC67B2-EE68-5E91-A8C2-6738E762BE04; **Taxon:** scientificName: *Otaciliatongboshan* sp. nov.; **Location:** country: China; stateProvince: Jiangxi; locality: Shangrao City, Guangfeng District, Tongboshan National Natrue Reserve, near Dadongkeng Nature Reserve Protection Station; verbatimElevation: 827 m; verbatimCoordinates: 28°07'55.37"N, 118°12'22.90"E; georeferenceProtocol: GPS; **Event:** samplingProtocol: sieving; eventDate: 30/11/2024
- **Type status:** Paratype. **Occurrence:** recordedBy: Liu Ke-Ke; individualCount: 3; sex: female; lifeStage: adult; occurrenceID: 9B3B8F77-7BD1-516A-8F3F-6A5170A6A80F; **Taxon:** scientificName: *Otaciliatongboshan* sp. nov.; **Location:** country: China; stateProvince: Jiangxi; locality: Shangrao City, Guangfeng District, Tongboshan National Natrue Reserve, near Dadongkeng Nature Reserve Protection Station; verbatimElevation: 827 m; verbatimCoordinates: 28°07'55.37"N, 118°12'22.90"E; georeferenceProtocol: GPS; **Event:** samplingProtocol: sieving; eventDate: 30/11/2024
- **Type status:** Paratype. **Occurrence:** recordedBy: Liu Ke-Ke; individualCount: 1; sex: female; lifeStage: adult; occurrenceID: 55EC586D-5124-5D7F-99F4-730A71AA82D5; **Taxon:** scientificName: *Otaciliatongboshan* sp. nov.; **Location:** country: China; stateProvince: Jiangxi; locality: Shangrao City, Guangfeng District, Tongboshan National Natrue Reserve, Shuangxikou; verbatimElevation: 321 m; verbatimCoordinates: 28°07'54.93"N, 118°15'44.14"E; georeferenceProtocol: GPS; **Event:** samplingProtocol: sieving; eventDate: 1/12/2024
- **Type status:** Paratype. **Occurrence:** recordedBy: Liu Ke-Ke; individualCount: 3; sex: male; lifeStage: adult; occurrenceID: B3DF0013-806B-5601-8CF2-120A38037102; **Taxon:** scientificName: *Otaciliatongboshan* sp. nov.; **Location:** country: China; stateProvince: Jiangxi; locality: Shangrao City, Guangfeng District, Tongboshan National Natrue Reserve, Fengjinshan; verbatimElevation: 493 m; verbatimCoordinates: 28°07'02.46"N, 118°16'57.80"E; georeferenceProtocol: GPS; **Event:** samplingProtocol: sieving; eventDate: 2/12/2024
- **Type status:** Paratype. **Occurrence:** recordedBy: Liu Ke-Ke; individualCount: 9; sex: female; lifeStage: adult; occurrenceID: B84FD5CC-615F-5F75-9C7C-135EB9FB9FB2; **Taxon:** scientificName: *Otaciliatongboshan* sp. nov.; **Location:** country: China; stateProvince: Jiangxi; locality: Shangrao City, Guangfeng District, Tongboshan National Natrue Reserve, Fengjinshan; verbatimElevation: 493 m; verbatimCoordinates: 28°07'02.46"N, 118°16'57.80"E; georeferenceProtocol: GPS; **Event:** samplingProtocol: sieving; eventDate: 2/12/2024
- **Type status:** Paratype. **Occurrence:** recordedBy: Liu Ke-Ke; individualCount: 1; sex: female; lifeStage: adult; occurrenceID: 9D761191-AB6A-5F93-8491-687F1F5A86B9; **Taxon:** scientificName: *Otaciliatongboshan* sp. nov.; **Location:** country: China; stateProvince: Jiangxi; locality: Shangrao City, Guangfeng District, Tongboshan National Natrue Reserve, Damenkeng; verbatimElevation: 405 m; verbatimCoordinates: 28°09'43.78"N, 118°15'23.32"E; georeferenceProtocol: GPS; **Event:** samplingProtocol: sieving; eventDate: 3/12/2024
- **Type status:** Paratype. **Occurrence:** recordedBy: Liu Ke-Ke; individualCount: 3; sex: male; lifeStage: adult; occurrenceID: 0318EDF2-B37F-57D9-8DE5-F8643157FFF0; **Taxon:** scientificName: *Otaciliatongboshan* sp. nov.; **Location:** country: China; stateProvince: Jiangxi; locality: Shangrao City, Guangfeng District, Tongboshan National Natrue Reserve, middle section of Damenkeng; verbatimElevation: 364 m; verbatimCoordinates: 28°09'34.09"N, 118°15'13.90"E; georeferenceProtocol: GPS; **Event:** samplingProtocol: sieving; eventDate: 3/12/2024
- **Type status:** Paratype. **Occurrence:** recordedBy: Liu Ke-Ke; individualCount: 3; sex: female; lifeStage: adult; occurrenceID: C9419E61-07B6-51B8-B73A-50A278B8FA41; **Taxon:** scientificName: *Otaciliatongboshan* sp. nov.; **Location:** country: China; stateProvince: Jiangxi; locality: Shangrao City, Guangfeng District, Tongboshan National Natrue Reserve, middle section of Damenkeng; verbatimElevation: 364 m; verbatimCoordinates: 28°09'34.09"N, 118°15'13.90"E; georeferenceProtocol: GPS; **Event:** samplingProtocol: sieving; eventDate: 3/12/2024

#### Description

**Figure 1.**
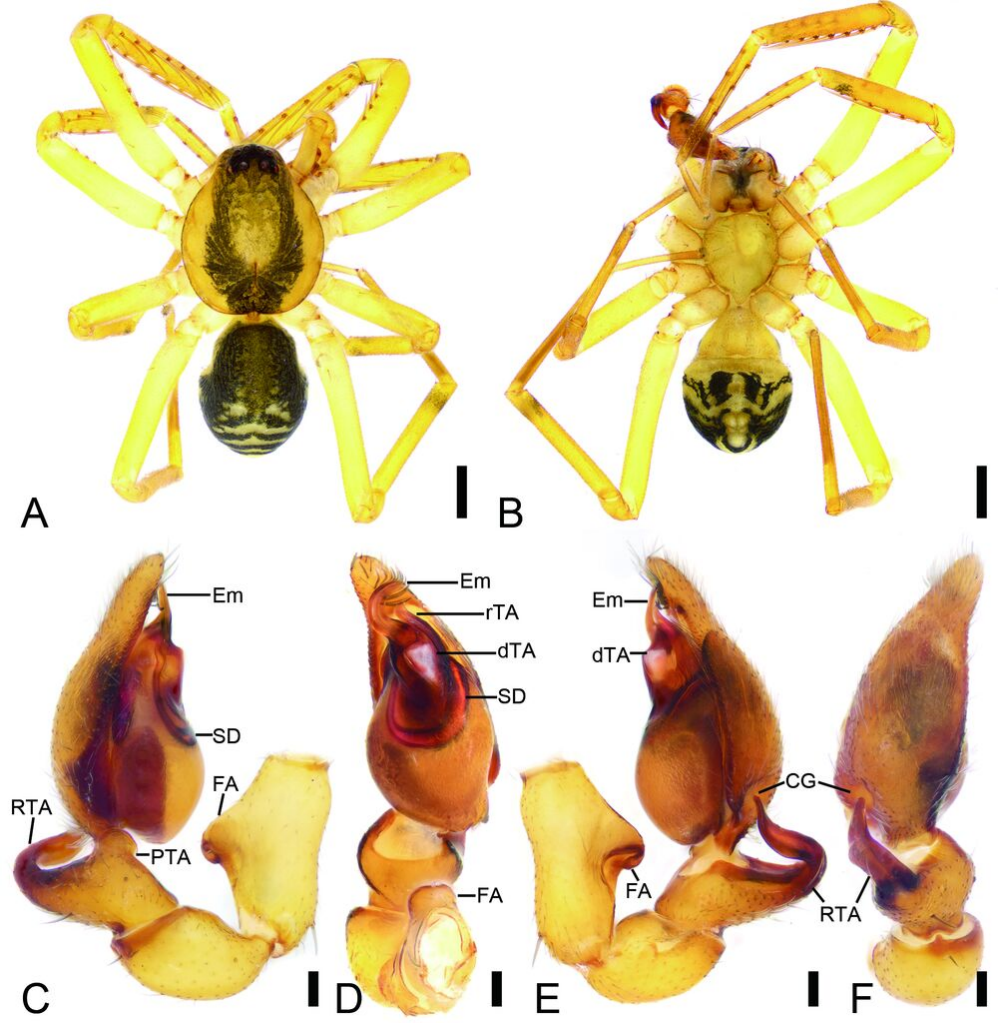
*Otaciliatongboshan* sp. nov., male holotype. **A** Habitus, dorsal view; **B** Same, ventral view; **C** Palp, prolateral view; **D** Same, ventral view; **E** Same, retrolateral view; **F** Same, dorsal view. Abbreviations: CG – cymbial groove, dTA – distal tegular apophysis, Em – embolus, FA – femoral apophysis, PTA – prolateral tibial apophysis, rTA – retrolateral tegular apophysis, RTA – retrolateral tibial apophysis, SD – sperm duct. Scale bars: 0.5 mm (A, B), 0.1 mm (C–F).

Male (Holotype). Habitus as in Fig. 1A and B and Fig. 4C and D. Total length 3.22, carapace 1.66 long, 1.43 wide.

Eye sizes and interdistances: AME 0.07, ALE 0.07, PME 0.07, PLE 0.06; AME–AME 0.05, AME–ALE 0.02, PME–PME 0.12, PME–PLE 0.06, AME–PME 0.11, AME–PLE 0.16, ALE–ALE 0.23, PLE–PLE 0.39, ALE–PLE 0.10. MOA 0.26 long, frontal width 0.38, posterior width 0.49. Chelicerae with three promarginal and six retromarginal teeth. Sternum (Fig. 1B), nearly as long as wide, posterior end blunt. Leg measurements: I 5.73 (1.84, 0.4, 1.44, 1.38, 0.67); II 5.01 (1.56, 0.44, 1.25, 1.1, 0.66); III 3.67 (1.13, 0.28, 0.75, 1.01, 0.5); IV 4.84 (0.98, 0.34, 1.24, 1.52, 0.76). Leg spination (Fig. 1A, B): femora I d11, p1111, II d1, p111, III d1, IV d1; tibiae I v22222222, II v2222222; metatarsi I v2222, II v1222. Pedicel 0.13 long. Abdomen (Fig. 1A and B) 1.43 long, 1.14 wide, dorsal scutum covering more than 1/2 length of abdomen.

Colouration (Fig. 1A, B). Carapace yellow with conspicuous, irregular, dark yellow mottled markings radially along mid-line and arc-shaped dark stripes around margin. Chelicerae, endites and labium yellow. Sternum yellow, with yellow brown lateral margins. Legs yellow, with a few stripes on tibiae and metatarsi. Abdomen brown, with pair of small round yellow-brown and pair of large oval yellowish spots on medial dorsal scutum, three pale chevron-shaped stripes on sub-posterior part and one yellowish arc-shaped stripe posteriorly; venter with the symmetrical black patterns on the posterior half.

Palp (Fig. 1C−F and Fig. 3A−F). Femoral apophysis (FA) well-developed, nearly as wide as half of femoral maximum width. Tibia with two apophyses: a large, thick, very strong retrolateral apophysis (RTA), longer than tibia, strongly bent inwards towards concaved cymbial groove (CG), with a slightly curved tip and one relatively broad, ridge-like prolateral apophysis (PTA). Sperm duct (SD) O-shaped in ventral view, reaching medial part of tegulum. Retrolateral tegular apophysis (rTA) clavate, thick, directed retrolaterally. Distal tegular apophysis (dTA) sub-triangular, anterior part broader than posterior, membranous, arising from base of embolus and retrolateral part of sperm duct. Embolus (Em) hook-shaped, strongly curved, with a broad base and very narrow groove (EG).

**Figure 2.**
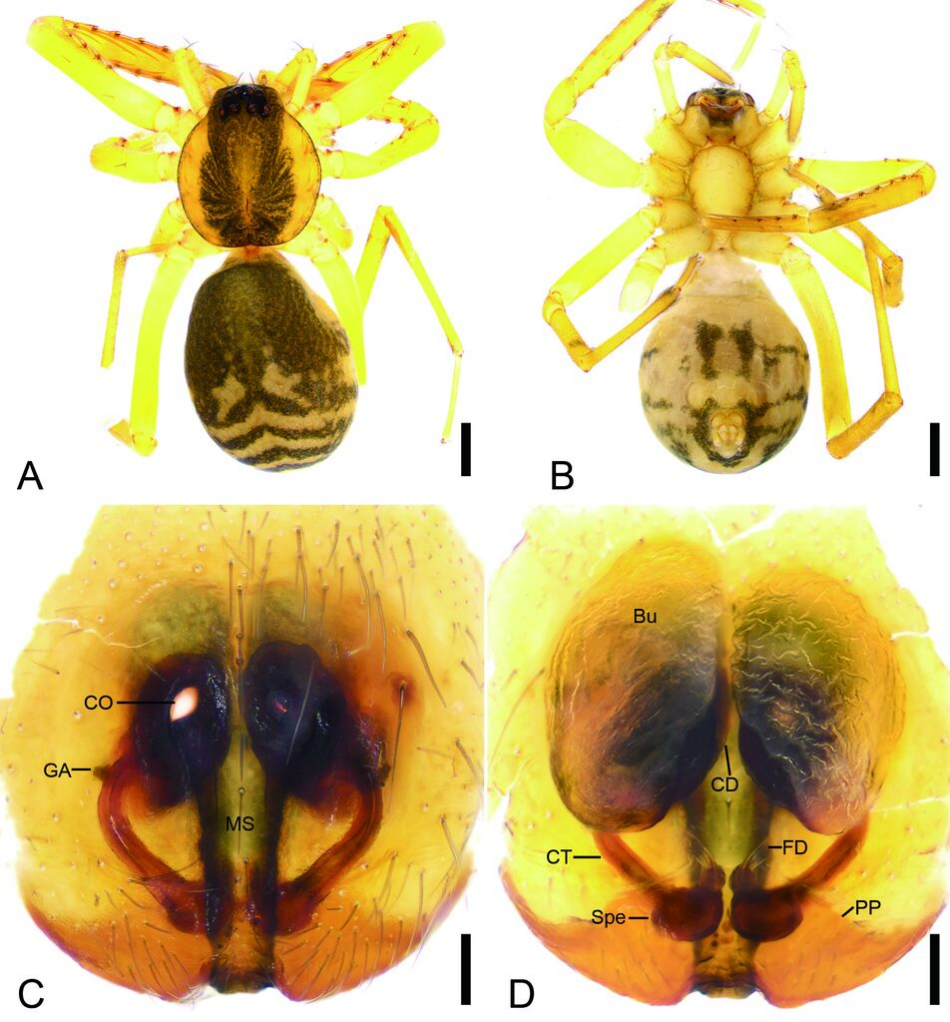
*Otaciliatongboshan* sp. nov., female paratype. **A** Habitus, dorsal view; **B** Same, ventral view; **C** Epigyne, ventral view; **D** Vulva, dorsal view. Abbreviations: Bu – bursa, CD – copulatory duct, CO – copulatory opening, CT – connecting tube, FD – fertilisation ducts, GA – glandular appendage, MS – median septum, PP – posterior plate, Spe – spermathecae. Scale bars: 0.5 mm (A, B), 0.1 mm (C, D).

**Female** (Paratype). Habitus as in Fig. 2A and B and Fig. 4E and F. Total length 4.13, carapace 1.59 long, 1.41 wide. Eye sizes and interdistances: AME 0.09, ALE 0.1, PME 0.08, PLE 0.09, AME–AME 0.05, AME–ALE 0.02, PME–PME 0.1, PME–PLE 0.06, AME–PME 0.08, AME–PLE 0.15, ALE–ALE 0.23, PLE–PLE 0.36, ALE–PLE 0.08. MOA 0.28 long, frontal width 0.39, posterior width 0.51. Leg measurements: I 6.2 (1.76, 0.47, 2, 1.39, 0.58); II 4.81 (1.37, 0.42, 1.39, 1.09, 0.54); III 3.69 (1.21, 0.24, 0.65, 1.08, 0.51); IV 5.48 (1.66, 0.42, 1.08, 1.81, 0.51). Leg spination (Fig. 2A and B): femora I d11 p1111, II d1 p111, III d1, IV d1; tibiae I v22222222, II v2222222, metatarsi I v2222, II v1222. Pedicel 0.06 long. Abdomen (Fig. 2A and B) 2.48 long, 1.65 wide.

Colouration (Fig. 2A and B). Paler than male.

**Figure 3.**
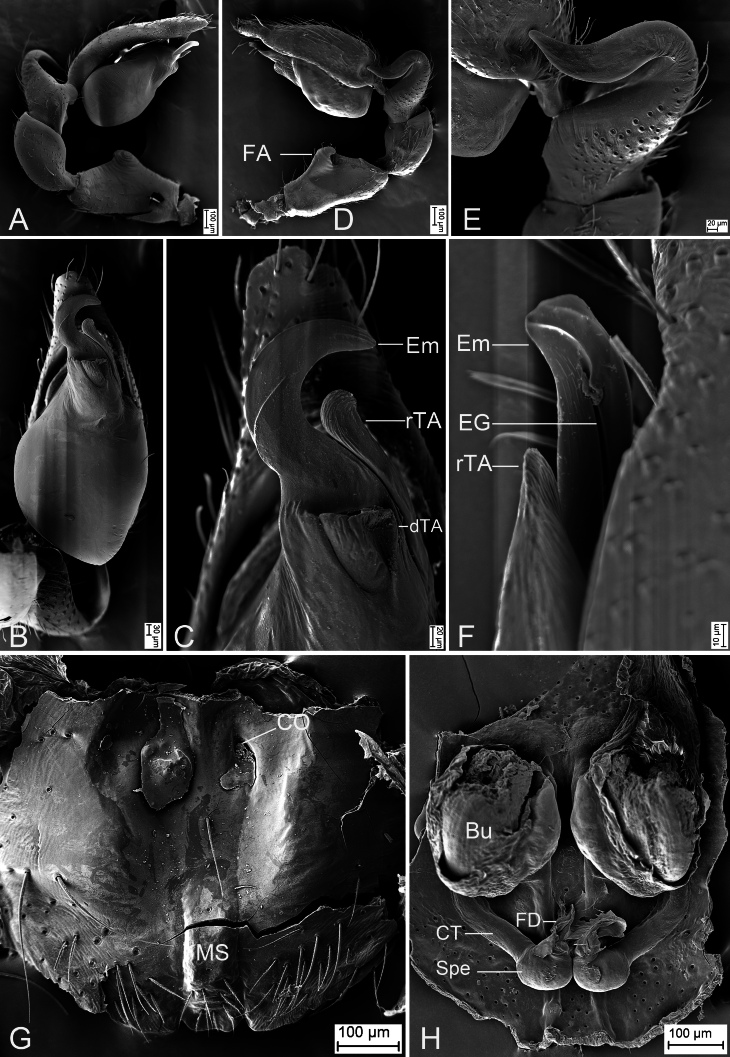
*Otaciliatongboshan* sp. nov., male palp and female epigyne, paratype. **A** Palp, prolateral view; **B** Same, ventral view; **C** Same, detail of Em, rTA and dTA, ventral view; **D** Same, retrolateral view; **E** Same, detail of RTA, retrolateral view; **F** Same, detail of Em, EG and rTA, retrolateral view; **G** Epigyne, ventral view; **H** Same, dorsal view. Abbreviations: Bu − bursa, CO − copulatory opening, CT − connecting tube, dTA − distal tegular apophysis, EG – embolic groove, FA − femoral apophysis, FD − fertilisation ducts, MS − median septum, rTA − retrolateral tegular apophysis, RTA − retrolateral tibial apophysis, Spe − spermathecae.

Epigyne (Fig. 2C and D and Fig. 3G and H). Epigynal plate trident-like through integument, median septum (MS) sub-rectangular, anterior part slightly broader than posterior. Copulatory openings (CO) anteromedially located, oval, separated by almost the width of median septum. Posterior plate (PP) slightly sclerotised, medially concaved, covering spermathecae. Copulatory ducts (CD) relatively broad, posteriorly with pair of bursae laterally. Bursae (Bu) large, soybean-shaped, transparent, slightly touching, nearly covering 2/3 of valvae, with a slightly sclerotised base. Glandular appendages (GA) very short, indistinct in ventral view, mastoid-like through integument in ventral view, located on posterior of copulatory ducts. Connecting tubes (CT) thin, convergent from medio-lateral to subposteromedial part of vulva. Spermathecae (Spe) globular, slightly separated. Fertilisation ducts (FD) short, with a small spherical base, located at anterior of spermathecae, directed anterolaterally.

#### Diagnosis

The male of the new species is similar to *Otaciliaguizhumao* Liu & Li, 2022 in having a hook-shaped embolus and a thick, clavate retrolateral tegular apophysis (see Liu et al. (2022): 354, Suppl. 2: 54, fig. 82), but can be separated from it by the carapace with broad dark yellow mottled markings radially along mid-line (vs. dark brown markings radially on the surface) (cf. Fig. 1A and Liu et al. (2022): fig. 82A), the thick (vs. moderate) and very strong (vs. moderate) bending retrolateral tibial apophysis (cf. Fig. 1E and Liu et al. (2022): fig. 82E) and the subtriangular distal tegular apophysis (vs. oval) (cf. Fig. 1D and Liu et al. (2022): fig. 82D). The females resemble that of *O.guizhumao* in having the oval copulatory openings, the large, soybean-shaped, slightly separated bursae nearly covering 2/3 of vulvae, the thin convergent connecting tubes and the globular, slightly separated spermathecae (see Liu et al. (2022): 354, Suppl. 2: 54, figs. 84C and D), but can be distinguished from it by the carapace with broad dark yellow mottled markings radially along mid-line (vs. dark brown markings radially on the surface) (cf. Fig. 2A and Liu et al. (2022): fig. 84A) and the relative thin median septum (vs. broad) (cf. Fig. 2C and Liu et al. (2022): fig. 84C).

#### Etymology

The species name is derived from the name of the type locality; noun in apposition.

#### Distribution

Known only from the type locality in Jiangxi Province, China (Fig. 5). This species has a wide distribution in Tongboshan National Nature Reserve, based on fieldwork.

#### Biology

**Figure 4.**
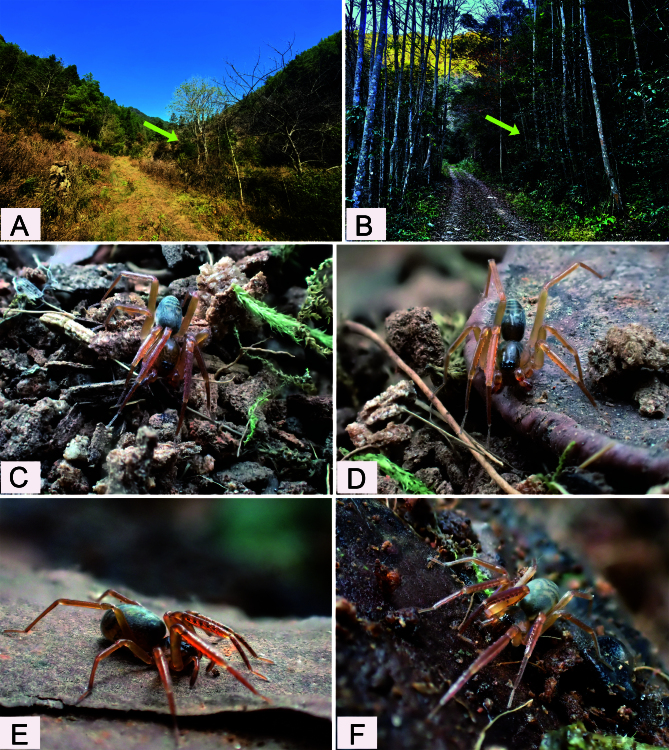
*Otaciliatongboshan* sp. nov. **A, B** Habitat, yellow arrows show the sampling points; **C, D** Male; **E, F** Female.

**Figure 5.**
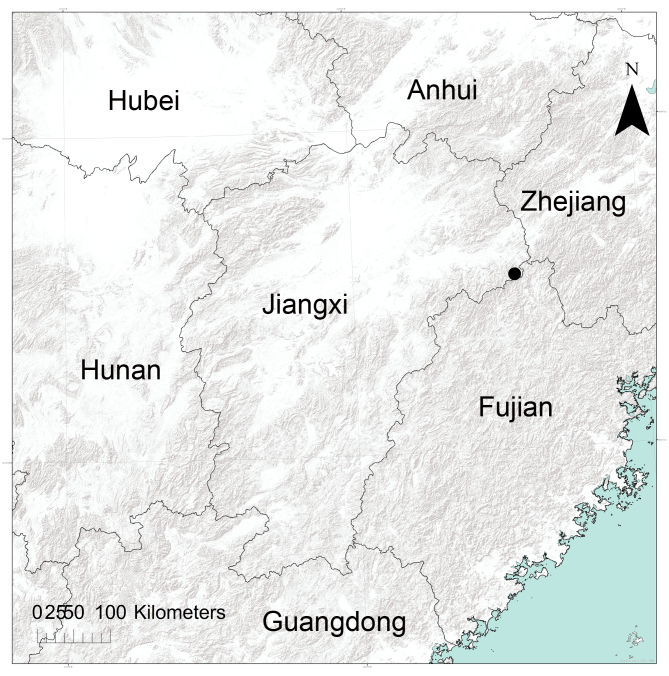
The location of the Tongboshan National Nature Reserve in Jiangxi Province, China indicated by a large black dot.

It was collected from leaf litter by using the seiving method in areas of broad-leaved forests (Fig. 4A and B) in hilly areas.

## Supplementary Material

XML Treatment for Otacilia tongboshan
